# Determinants of parental traditional medicine use for children during COVID-19 in Dire Dawa city administration, Eastern Ethiopia, 2023/24: Mixed community based cross-sectional study design

**DOI:** 10.1371/journal.pone.0354889

**Published:** 2026-07-30

**Authors:** Mohammed Kebede, Gudeta Kusheta, Mehammedamin Jemal, Kedir Abdurehman, Netsanet Melkamu, Yakob Tadese

**Affiliations:** 1 Department of Nursing, College of Medicine and Health Sciences, Dire Dawa University, Dire Dawa, Ethiopia; 2 Department of Pediatrics and Child Health Nursing, School of Nursing, College of Medicine and Health Sciences, Wolaita Sodo University, Wolaita sodo, Ethiopia; Endeavour College of Natural Health, AUSTRALIA

## Abstract

**Introduction:**

Globally, 85% of the population uses traditional medicine. Ethiopia is a country with various traditional medicinal practices. However, various studies also reported traditional medicine used in children by their parents has had adverse outcomes and hospitalized them with complications. Various studies had also reported that traditional medicine use was high during COVID-19. Despite the fact that, children are particularly vulnerable population, limited studies exists regarding the prevalence, types, and determinants of parental TM use during COVID-19. In addition the existing studies were also confined to quantitative methods. Therefore, this study uses a mixed study design to explore indigenous methods of traditional medicine knowledge acquisition, preservation, preparation, storage, and dosage of herbs; barriers of disclosure; and prevalence of concomitant use.

**Methods:**

A community-based concurrent mixed study design was conducted among 941 randomly selected parents from Dec 01/2023 to March 01/2024. The data was cleaned, coded, and entered in Epi-Data version 4.6 and transferred to Stata version 14.1 for analysis. Finally, an AOR with a 95% CI was computed, and variables with a p-value < 0.05 in the multivariable analysis were taken as significant factors. The qualitative data was collected from 16 selected key informants by in-depth face-to-face interviews and analyzed by using thematic analysis.

**Result:**

The prevalence of parental TM use for children was 87.6%. Sibling relation to child (AOR: 0.45, 95% CI, 0.01–0.77), religion (AOR: 0.11, 95% CI, 0.04–0.29), parental TM use for themselves (AOR: 6.55, 95% CI, 1.67–25.69), easy accessibility (AOR: 2.72, 95% CI, 1.16–6.40), safety & efficacy (AOR: 4.53, 95% CI, 2.3–13.61), and TM skill in the family (AOR: 0.47, 95% CI, 0.19–0.84) were determinants of TM utilization. Lack of awareness, misperception of TM, lack of trust in HCP and negative judgment from HCP were the main-barriers for non-disclosure of TM use.

**Conclusion:**

This result indicated traditional medicine use was high; nearly 9 out of 10 parents had used TM for their children during COVID-19. There was high concomitant utilization (90.6%) and high non-disclosure rate (98.1%) of traditional medicine use to health workers. Hence, it is better to give priority to strengthening the implementation of TM policy and manage accordingly.

## Introduction

Traditional medicine (TM), also called indigenous, folk, or local medicine, is defined as a diverse set of health practices indigenous to different cultures used in the maintenance of health, as well as in the prevention, diagnosis, improvement, or treatment of physical and mental illnesses [1]. The World Health Organization (WHO) estimates four billion (80–85%) of the world’s population relies on TM either alone or in combination with conventional medicine [2]. The utilization ranges from 70–95% in developing countries specifically in Asia and Africa, whereas China, accounts around 40% of all health care delivery system [3]. Similarly, 80% of the Ethiopian population and up to 90.3% of parents depend on TM for their primary health care needs [4–6].

The history of TM is as long as the history of human beings; in almost every country, it remains either the mainstay of the health care delivery system or a complement [1]. Therefore, TM has played a significant role in human health; however, concerns about safety, quality, efficacy, preservation, and regulation remain major issues in many parts of the world [7]. Particularly in children, the risk of complications was high compared to adults [8]. This may be due to the difference in the physiologic and bioavailability variation of herb-drug [9] and lack of pragmatic consideration of their age and other health conditions during dosage [10,11].

Various studies reported traditional medicine used in children by their parents has had adverse outcomes and hospitalized them with complications [12–14]. TM results in a wide range of complications, from mild allergic reactions and gastrointestinal issues to severe outcomes such as respiratory problems, blood clotting disorders, liver toxicity, renal failure, and multi-organ dysfunction syndrome [14,15]. Among children who receive invasive traditional herbal medicine, approximately 30.96% develop multi-organ dysfunction syndrome; over 25% of children admitted to the pediatric intensive care units were suspected of experiencing toxicity (30, 31), and nearly one-third of them died in intensive care units [15–18]. Easy availability with inadequate information on drug-herb interaction and high concomitant use may increase the risk of unwanted interactions [19–21]. Additionally, a study has reported more than half of all TMs are prescribed, dispensed, and sold illegally by traditional healers and parents and used incorrectly [10]. Furthermore, a high proportion (55–100%) of parents do not disclose their use to a health care provider [22,23]. Finally, they may end up with unexpected toxicities and possible under-treatment that results in various complications and poor treatment outcomes [24,25].

In recent decades, the increasing demands of TM services across Africa had attracted the attention of the WHO in advocating the integration of TM with modern health care systems [22]. This in turn initiates the implementation of various strategies in Africa through policy formation, research promotion, and inclusion of TM courses into curricula of healthcare training institutions in countries [22,26]. In spite of such progress, African countries such as Ethiopia continue to struggle with an absence of TM policy or its implementation, inadequate TM research infrastructure, and insufficient regulation of TM products and practices [6,27].

Above all, during the COVID-19 pandemic, TM use overwhelmed the healthcare delivery system across the world [28]. Even those countries with well-established health systems, such as China, India, and Japan, had doctors and scientists recommending TM as their mainstay for the treatment of COVID-19 [29–31]. The influence of such countries, coupled with media campaigns, and the unavailability of vaccines and treatment, coupled with a weak African health sector, makes the community rely on indigenous knowledge and new alternatives [32,33].

Various studies reported parental utilization of TM was associated with Covid-19 [32], low income [34], educational status [35], affordability and easy availability of practitioners [19,20]. In addition the misconception that herbs are superior to synthetic drugs, the high cost and parents’ fear of side effects of modern drugs were factors associated with TM use [35,36].

Even though children are the most vulnerable group, very limited research has been conducted on the prevalence, types, and determinants of parental TM use during COVID-19 in Ethiopia. More importantly, the existing studies were confined to quantitative methods. Therefore, this study used a mixed study design that would provide in-depth insight on indigenous parental TM use during COVID-19 and other important variables that would assist policymakers, researchers, and other concerned bodies.

## Materials and methods

### Study design and setting

A Community based concurrent mixed study design was implemented; employing concurrent triangulation strategy to assess the determinants of parental traditional medicine use for children during Covid-19, at Dire Dawa, Eastern Ethiopia. The study was conducted from December 01/2023 – March 01, 2024. Dire Dawa located 515kilometer away from Addis Ababa, the capital city of Ethiopia. According to latest unpublished health bureau data of July1, 2019 the current total population of the city was 492,644. It has 9 urban Kebeles (Smallest administrative unit of Ethiopia) with population of 325,440 (66%) in 80,097 households (HH) and 38 rural Kebeles classified under four cluster comprising 34,123(44%) population living in 167,200 household. The administration has 7 hospitals (3 governmental, 2 private, 2 other non-governmental, and 1 other governmental hospital) l and 57 health facilities (38 health posts, 15 health centers, and 4 private hospitals).

### Population and sample

For the quantitative study, the source population includes all parents children under 18 year in Dire Dawa city administration. The study population incorporates parents of children resides within randomly selected households during study period in Dire Dawa Ethiopia, 2023/24. Parents residing with their children under 18 years of age during data collection period were included whereas those who were absent excluded from the study. For the qualitative study, the source population includes key informants (TM healers/practitioners) and purposefully selected parents in Dire Dawa city administration. Accordingly the study population includes purposefully selected key informants and parents of children during study period in Dire Dawa Ethiopia, 2023/24.

### Sample size determination and sampling procedure

The sample size was determined using Epi- info version 7.2.5, considering double population proportion formula assumptions: Z α/2 = 95%, CI = 1.96, ZB = 90% power and r is the non-exposed to the exposed ratio (1:1). To estimate the sample size, information on significant factors taken from a study conducted on the prevalence and factors associated with parental use of TM for children at Motta town, Amhara Regional State [6], and Tole district, Oromia Region [37] (Table 1).

**Table 1:** Sample size determination for parental TM use for children 2023/24.

Variables	Proportion		Sample size	Final sample size after adding non response rate of 10%
P1	P2
Accessibility of TM	92.8	84	458(43)	503
Cheap Price of TM	93.1	85.6	582(43)	640
Effectiveness of TM	92.8	84	470(43)	517
Duration of illness	88.5	70	392(44)	431

Where: P1 = is the percent of exposed with the outcome, P2 = is the percent of non-exposed with the outcome, Z α/2 = is taking 95% CI, which is 1.96 ZB = 90% power and r is the non-exposed to the exposed ratio (1:1)

Therefore, the required sample size is assumed to be the largest one, which is n = 582. Finally considering design effect to adjust the variation and adding a 10% non-response the final sample size becomes **960. Whereas, sample** size for the qualitative data was determined by saturation of data.

**Fig 1:**
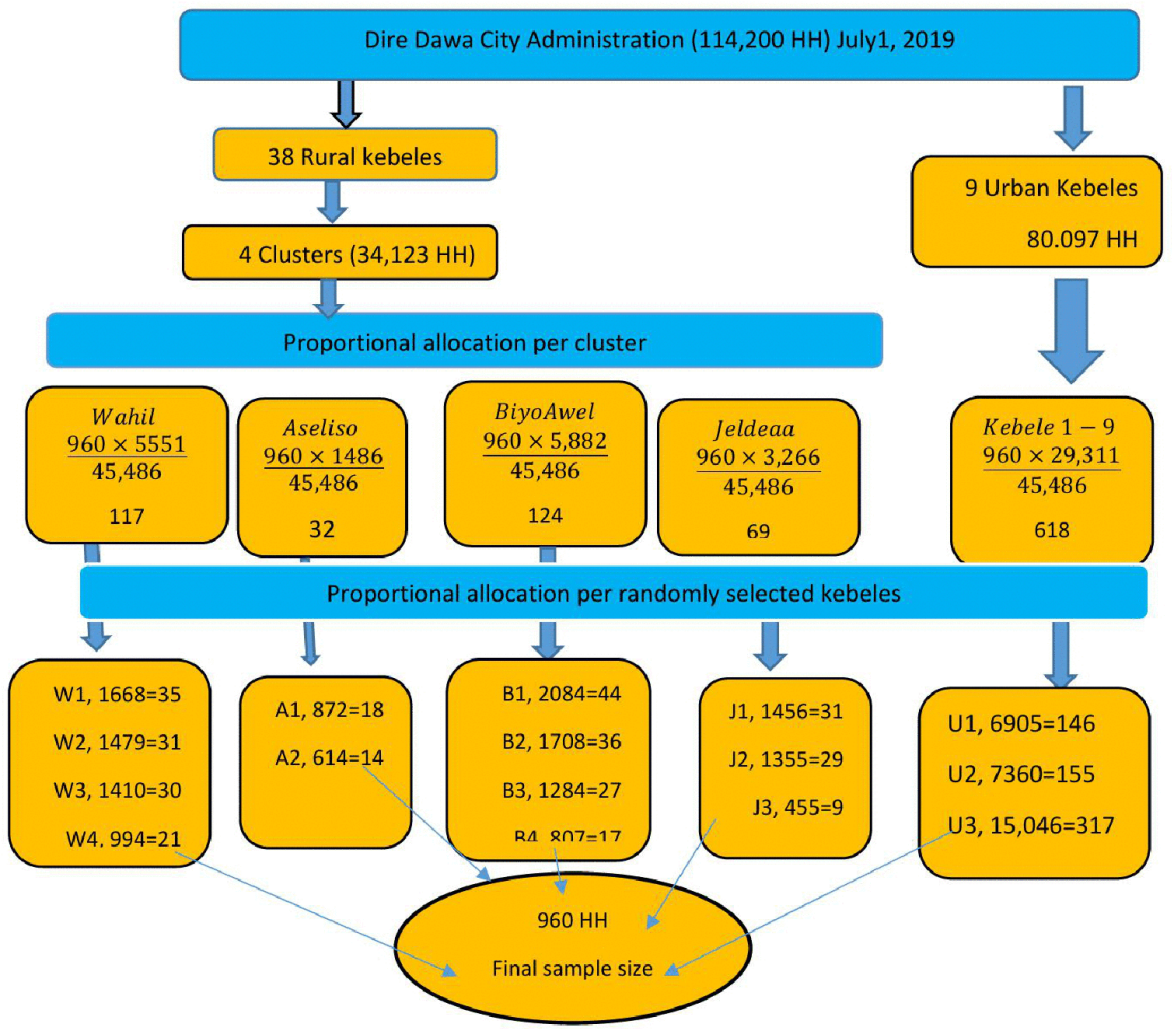
Schematic representation of sampling procedure and technique for quantitative study to assess determinants of parental TM use for children during Covid-19 in Dire Dawa city, Eastern Ethiopia, 2023/24.

To select study participants a multi-stage sampling technique was used from a total of 114,200 HH in 9 urban and 38 rural kebeles. Rural kebeles are further divided in to four clusters. To include kebeles from all clusters random sampling was purposefully used per the clusters, then 16 study kebeles was selected and finally proportional allocated based on their study population. (Fig 1)

Key: Randomly selected kebeles under each cluster with their respective HH & proportionally allocated sample

W (Wahil Cluster): W1 = Lega Oda Mirga, W2 = Jelo Belina W3 = Wahil kebele W4 = Hulu Mojo

A (Aseliso Cluster):A1=Hula Hulul A2=Gende Rige

B (Biyo Awale Cluster):B1=Awale cluster B2=Adadda B3= Beke Hallo B4=Jeaneni

J (Jaldesa Cluster):J1=Jaldesa kebele J2=Chire Miti J3=Legedini

U (Kebeles From Urban Cluster):U1=Kezira U2=Gende Kore U3=Gende Gereda

### Data collection tools and technique

Data were collected using a structured questionnaire for the quantitative study and a semi-structured interview guide was used for the qualitative study that adapted after reviewing various study [22,23,38–40]. It was prepared in English, then translated into Amharic, Aft Somalia, Harari, and Afaan Oromo language, and then retranslated to English to check for consistency. In order to assess the validity and reliability of the instrument, clarity of the questions and respondent reaction to the question and interviewer, pre-test was done in another kebeles on 5% of actual respondents. After the pre-test unclear questions were collected and interviewers adjusted themselves as required. The study participants’ language fluency was asked and interviewed based on their choice. A total of 10-trained data collectors and five supervisors were recruited for data collection and supervision. Data collectors were trained for 3 days before data collection. Finally, the trained data collectors collected data using face to-face interview with semi-structured questionnaires. The data collection procedure for the quantitative data, illustrated as follows: first, eligible household was selected, and then caregiver of the children was selected and interviewed. During selection of the caregiver the priority was given to the mother, if the mother was not available, the father or the guardian was interviewed. For the parents who used more than one type of TM for their children, lastly used TM was selected for the study to minimize recall bias. In the qualitative part of the study, the investigators collected the data from selected key informant through in-depth face-to-face interview. During the data collection, the interviews was started using open-ended questions and, later probing questions was forwarded as far as more clarifications are needed. The interviewing processes was stopped when data saturations reached. It was collected after the completion of quantitative data collection. A minimum of 30 minutes and a maximum of 60 minutes was used to complete the interview. The whole interviews was audio recorded to ensure accuracy after explicit consent was obtained Personal notes was taken simultaneously in quiet and private place of participant home using the language parents communicate.

### Study variables and measurement

The dependent variable was parental TM use for children during Covid-19 (Yes/No), whereas the independent variables were Socio-demographic characteristics of parents (age, sex, religion, marital status, income, education, family size, and residency). Enabling Factors (Cost, accessibility, source of referral, and distance from the health facility, and member of community-based health insurance (CBHI), Need Factors (Purpose of TM use such as promotion, prevention, and treatment), duration of illness, type of illness, and parental rating of child health). Health Care Practice and Experience Related Factors (Level of satisfaction with TM, the effectiveness of TM, and Dissatisfaction and fear of side effects of modern medicine, disclosure of TM use inquiry about TM use by HCP).

### Operational definitions

**Traditional Medicine(TM):** Refers to any health practices, knowledge, and beliefs used in the promotion of health, prevention of illness and treatment of diseases and not currently considered to be part of modern medicine but accepted in that community. TM is neither prescribed by the healthcare professional nor used commonly as a diet in that particular culture.

**Herbal medicines:** are plants used for medical treatments, which are culturally acceptable.

**Traditional healers/practitioners:** A care providers who are not trained in modern medical science such as herbalist, bone settler, tooth extractors, trained traditional birth attendant, religious fathers

**Functional foods:** Foods that provide both physiological preventive and/or health-promoting effects to reduce the risk of chronic diseases and basic nutrition in the form of small solids or droplets. Functional foods include garlic, onion, ginger, orange juice, red pepper spice, pepper, to mention but a few.

**Religious/prayer therapy:** are used in counseling to invite God’s healing presence to come and restore, forgive, erase, transform, and set free the inner life of the client to allow him or her to detach from sinful choices and painful trauma, and grow in all that Christ would have. Tsefat/Kitab, tselot and Tsebel can be cited as examples in the area.

**Massage:** Practice of applying gentle or strong pressure to the muscles and joints of the body to ease pain and tension using the hand that is carried out by a locally reputable healer.

**Bone settler:** Traditional practitioner who cures the balance of the skeleton, muscles and joint manipulation. He/she educates his/herself from tradition and takes up the practice of healing without having had any formal training.

**Perception of health status of the child:**

Poor: bed ridden and mental illness

Good: the child had some symptom disease

Very good: the child had not any sign and symptom of a disease

**Parent:** Father, mother or/and guardian who nurtures and raises a child.

**Children:** For the purpose of this study, children are those who are below 18 years

### Ethical approval and consent to participate

Ethical clearance was obtained on 5/15/2023 from the Dire Dawa University Institutional Review Board in a letter protocol number DDU-IRB-2023–187. Official letters of cooperation were written from DDU to Dire Dawa Administration Health Bureau (DDAHB). Permission letters were also be obtained from DDAHB. A formal letter of cooperation from the regional health bureau was submitted to the respective Kebeles administrative body for data collection. Consent from the parents of the children and assent from the adolescent was obtained. The obtained data was not disclosed to a third person despite study team and kept confidentially. Data was coded and locked by password. Any name and/or other personal information with respect to study subject was not used. Upon getting the necessary data, an acknowledgment was forwarded to the respective body.

The participants were assured the right to refuse or withdraw at any time. Finally, voluntary written and signed (thumbprint for those illiterate or unable to signed) informed consent was obtained from the study participant or their parents/legal guardians before data collection. The Confidentiality of the study participants was maintained throughout the research process by giving the code for participants. The study participants were also informed that data were kept private and confidential, and only used for research purposes.

### Data quality control and management

To ensure quality of data tool translated into the local languages Amharic, Af Somalia, Harari, and Afaan Oromo. The data collectors were recruited based on their language fluency in Amharic, Af Somalia, Harari, and Afaan Oromo. The recruited data collectors trained for two days on the objective, confidentiality of information, informed consent, and techniques of interviews. Five supervisors supervised the data collectors and reported daily. Before data collection, pretest was conducted on 5% of the total sample and modified accordingly. The collected data checked for completeness and consistency on the day of data collection. Simple frequencies and cross-tabulation were done for missing values and cross-checked with hard copies of the collected data.

For the qualitative data: The interview guide was pre-tested on four parents and two key informant in a non-selected kebeles. Feedback from the pilot was used to refine the question clarity, sequence and cultural appropriateness. In-depth interviews was transcribed verbatim in Amharic/Afaan Oromo/Af Somalia audios and then translated into the English language. The Data was analyzed using thematic analysis approach. Each transcript was carefully screened and triangulated with the quantitative data. Silent environment was used during data collection. Furthermore peers reviewed the transcription, translation and coding of the data. In ensuring trust worthiness four basic criteria of qualitative study were maintained through ensuring its credibility, dependability, transferability and conformability. In ensuring credibility: Before starting the actual data collection, good communication environment with the participants was established. Participants was also advised to stay open and relaxed during the interview as to get the correct data. Member checking was also done. In ensuring dependability: The investigators coded an interview separately and latter compare for inter coder dependability. To ensure transferability: This was proven by giving readers references so that the result of the finding can be applied to the same or similar situations. Detailed description of the research situation and methods to allow the understanding of the study to other researchers was made. In ensuring conformability: An audit trail\ was done which was recording unique topics found during the interview, giving reason for merging codes together and explaining of the meaning of the themes words of the participants were used in writing the finding of the research

### Data analysis

For the quantitative data: Data clean up and cross-checked before analysis and entered to Epi data version 4.6, finally exported to Stata version 14.1 for analysis. The descriptive statistics computed to summarize the descriptive results and presented in texts, tables, and figures. The model fitness checked by the Hosmer-Lemeshow goodness of a fit test. A bi-variable and multivariable logistic regression model computed to identify factors associated with parental TM use during covid-19. Variables that showed statistical significance during bi-variable analysis at P-value ≤ 0.25 entered to multivariable logistic regression. Finally, the strength of association are taken with adjusted odds ratios (AOR) with a 95% Confidence interval at a p-value < 0.05.

For Qualitative data: Data collection and analysis was conducted simultaneously. The data was analyzed by using inductive thematic analysis methods. First, the interviews was transcribed and carefully read several times in order to obtain a general sense of the entire interview (familiarization). Then, the texts was divided into condensed meaningful units. Later, these condensed units was abstracted and labeled with codes. Following this stage, the codes was sorted into categories and then to subcategories (themes) based on their similarities and differences in the software. Finally, themes was formulated as their presentation of the latent content of the text

## Result

### Socio-demographic characteristics, for quantitative study

Among 960 sampled participants, a total of 941 study participants were involved in the study, making a response rate of 98.02%. Out of 941 respondents, 610 (64.8%) were in the age group of 30–39 years. Among the total study participants, the majority, 765 (81.3%), were mothers of the children. In addition, from the total 941 respondents, 682 (72.5%) were married, and almost half, 392 (42.3%), were Muslim religion followers.

Regarding the educational status of the study participants, 473 (50.3%) have secondary school educational status. 481 (51.1%) were governmentally employed in their occupation. A majority, 618 (65.7%), of the residents were urban residents. Moreover, from a total of study participants, 415 (44.1%) have a monthly income in the range of 5251–7800 ETB. Most of the parents, 601 (63.9%), have less than or equal to three children in their household (Table 2).

**Table 2:** Socio-demographic characteristics of the study participants for the study to assess determinants of parental TM use for children during COVID-19 in Dire Dawa city, Eastern Ethiopia, 2023/24.

Variable	Category	Frequency	Percentage (%)
Age of the child	< 5yr	211	22.4
	6-11yr	461	49
	12-17yr	269	28.6
Parental/guardian Age	20-29 30-39	163 610	17.3 64.8
	40-49	137	14.6
	>/ = 50	31	3.3
Relation of the parent/ guardian to the child	Mothers	765	81.3
	Father	133	14.1
	Sibling	43	4.6
Marital status	Single	48	5.1
	Married	682	72.5
	Divorced	112	11.9
	Widow	99	10.5
Religion	Orthodox	286	30.4
	Protestant	257	27.3
	Muslim	398	42.3
Educational status	Can’t read and write	87	9.2
	Primary(1–8)	95	10.1
	Secondary(9–12)	473	50.3
	Higher	286	30.4
Occupation	Government Employee	481	51.1
	Private Employee Daily Labor	368 55	39.1 5.8
	Others(specify)	37	3.9
Residency	Rural	323	34.3
	Urban	618	65.7
Monthly income	<3200	105	11.2
	3201-5250	243	25.8
	5251-7800	415	44.1
	>7801	178	18.9
Number of children below 18 years	. < 3	601	63.9
	3-5	340	36.1

Key: Other Student, NGO (Non governmental organization)

### Parental health care practice & experience

**Fig 2:**
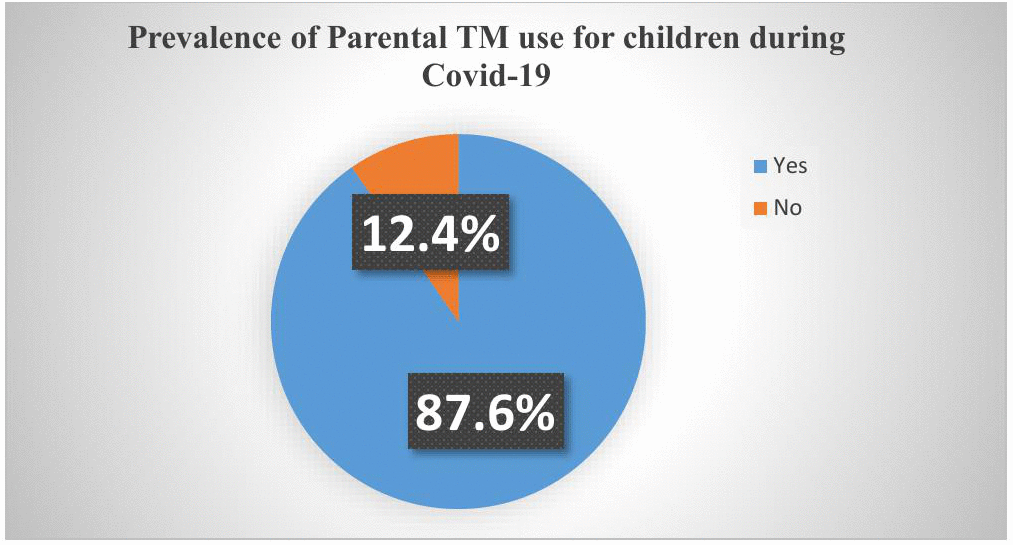
Prevalence of Parental TM use for children during COVID-19 in Dire Dawa city, Eastern Ethiopia, 2023/24.

Among the total 941 participants of this study, 824 (87.6%) had used TM for their children during the Covid-19 outbreak. Also 816 (86.7%) of the study participants had ever used TM for themselves (Fig 2).

**Fig 3:**
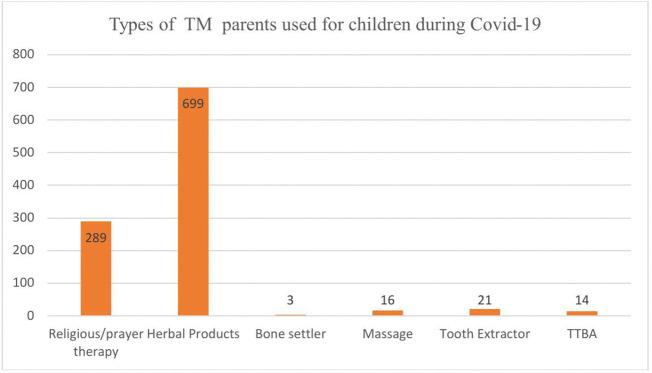
Types of TM parents used for children during COVID-19 in Dire Dawa city, Eastern Ethiopia, 2023/24.

Most of the parents, 699 (67.1%), used herbal products, followed by religious therapy, 289 (27.7%), for their children (Fig 3).

Regarding the household use of TM, it is used for almost all family members, and children and adults share the majority, 857 (44.9%), of the TM use. The majority, 708 (75.2%), of the TM was taken by oral route. Covid-19, 22 (1.6%), was the rare indication for parental TM use for their children, but respiratory illness, 324 (27.6%), was the major reason for parental TM use for children. Easily accessible and affordable, 480 (48.1%) was found to be the major reason why the parents prefer TM for children (Table 3).

**Table 3:** Parental health care practice and characteristics of the study participants to assess determinants of parental TM use for children during COVID-19 in Dire Dawa city, Eastern Ethiopia, 2023/24.

Variable	Category	Frequency	Percentage (%)
Have you ever used TM for yourself	Yes	816	86.7
	No	125	13.3
For whom TM is used in your family	Elderly people	466	24.4
	Children & adults	857	44.9
	Neonates & Infants	389	20.4
	Pregnant	196	10.3
Last time you applied TM for your child(n = 873)	Last week -month	22	2.5
	Month- Last six month	221	25.1
	6-12months	220	25.2
	A 1–2 years	259	29.7
	Before two years	151	17.2
Route TM applied	Oral	708	75.2
	Topical	197	21.1
	Rectal	35	3.7
Illness you preferred to use TM for your child	Covid-19	26	2.2
	Fever	16	1.3
	Headache	45	3.8
	Psychological	240	20.4
	Musculoskeletal	105	8.9
	Cardiac	162	13.8
	GIT	195	27.6
	Respiratory Dermatology	324 62	5.3
Reasons for preferring TM than modern medicine	Easily accessible & affordable	480	43.1
	Safety & efficacy	69	6.1
	Cheap in price	380	34.1
	Having low income Dissatisfaction with modern medicine Side effects fear of modern medicine Difficulty in accessing health care facilities	32 91 43 18	2.8 8.2 3.8 1.6

A majority, 677 (71.9%), of the parents also visited a health facility for the same illness after using TM due to no improvement by TM 616 (91%). 554 (81.8%) of the participants reported that the health care provider did not ask about pre-hospital TM usage. Similarly, almost all 530 (98.1%) of those parents who visited a health facility did not disclose their TM use for their children irrespective of the request by the health care provider. Concomitant use of 614 (90.6%) of TM and conventional drugs was also common among the study participants.

A majority, 511 (75.5%), of the study participants replied that it is unethical and illegal to discuss TM use for children with their health care provider. More than half, 556 (59.1%), of the participants stated that the TM provider did not tell them about the side effects of the TM. Moreover, almost half (486, 48.4%) of the study participants stated that TM has good efficacy, and the majority (635, 67.5%) of them recommend TM to be used by others (Table 4).

**Table 4:** Parental health care practice and experience characteristics of the study participants to assess determinants of parental TM use for children during COVID-19 in Dire Dawa city, Eastern Ethiopia, 2023/24.

Variable	Category	Frequency	Percentage
Visited health facility for same illness after using TM	Yes	677	71.9
	No	274	28.1
Reason for visiting	No improvement	616	91
	Develop side effect	61	9
Health care provider request about pre-hospital TM use	Yes	123	8.2
	No	554	81.8
Disclosure of TM use irrespective of HCP request	Yes	24	1.9
	No	530	98.1
Concomitant use	Yes	614	90.6
	No	63	9.4
Ethical and legal to discuss TM use for children with your health care provider?	Yes	166	24.5
	No	511	75.5
Provider of TM told you about the side effect of the TM	Yes	385	40.9
	No	556	59.1
Would you believe TM is side effect free?	Yes	339	36
	No	409	43.8
	Assumed to be side effect free	126	13.4
Level of satisfaction of TM use to your child	Completely dissatisfied Somewhat dissatisfied	143 138	15.1 14.6
	Neither satisfied nor	169	17.9
	dissatisfied	389	41.3
	Somewhat satisfied Completely satisfied	102	10.9
Level of efficacy of TM used	Excellent	316	33.6
	Good	456	48.4
	Poor	169	17.9
Do you recommend TM to be used for others?	Yes	635	67.5
	No	306	32.5

**Fig 4:**
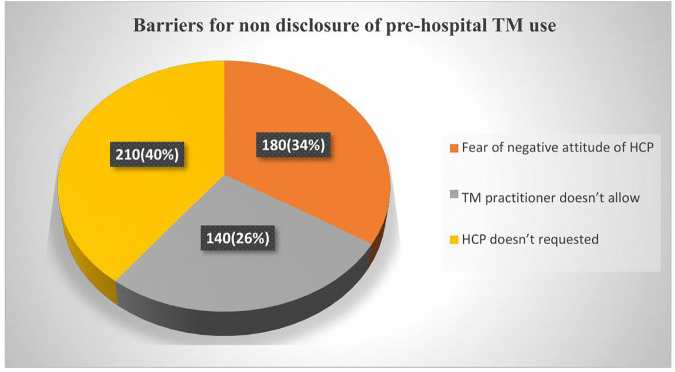
Barriers for non-disclosure of parental pre-hospital TM use to HCP during COVID-19 in Dire Dawa city, Eastern Ethiopia, 2023/24.

HCP does not requested 210(39.6%) was among the major factor for which the parents does not disclose pre-hospital TM use for their children during health care visit followed by fear of negative attitude of the HCP about the pre-hospital TM use (Fig 4).

### Enabling Factors

**Fig 5:**
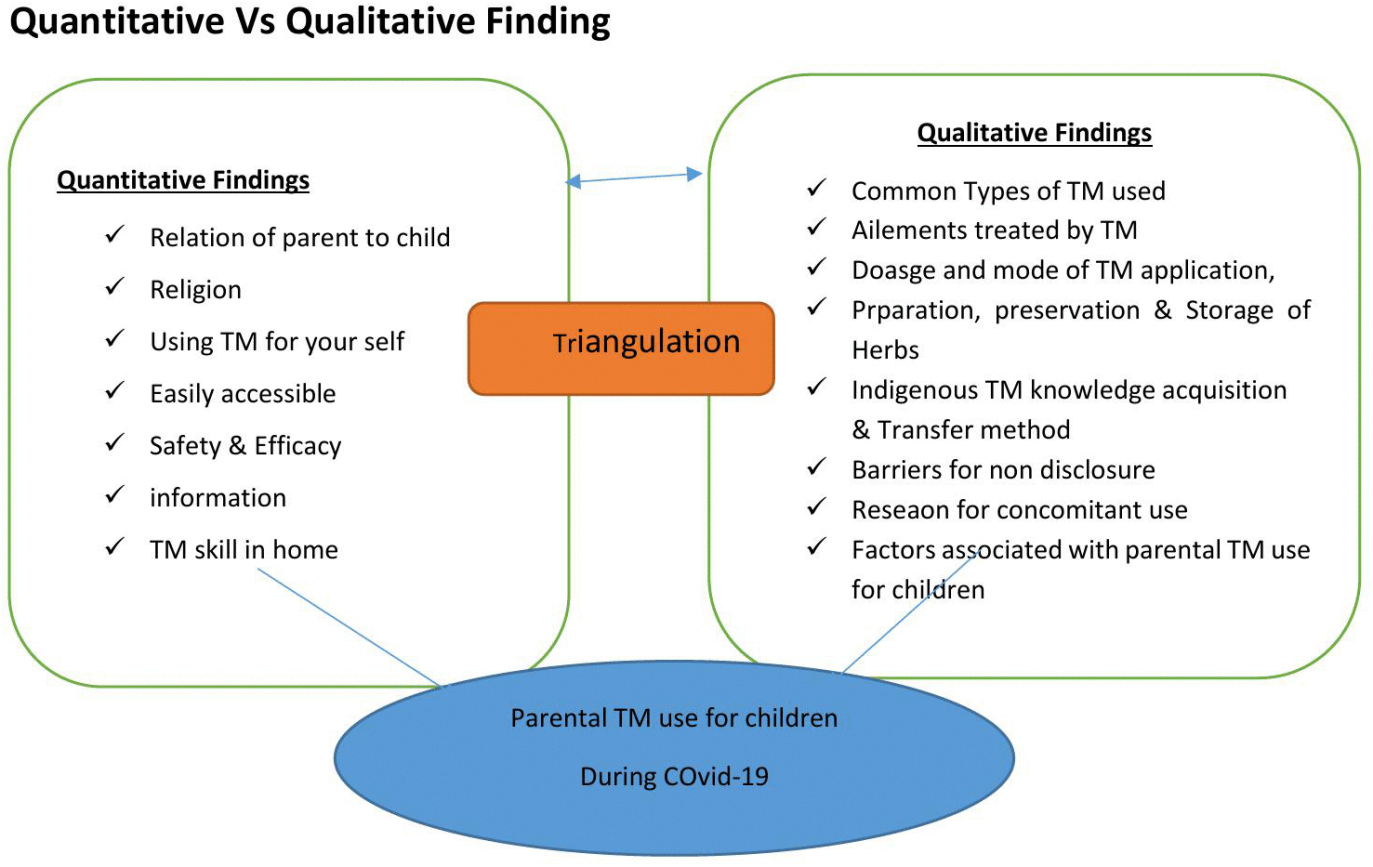
Triangulation of quantitative and qualitative findings.

Parental source of the information about TM for their children for most of the parents 703(38.9%) were family members, relatives, friends and neighbors. However, half of the parents 584(50%) get the TM for their children from traditional healers. Majority of the study participants 823(87.5) responded that they do not have a family member with TM skill. 676(71.8) of the parents had community based insurance (Table 5) (Fig 5).

**Table 5:** Enabling factors of the study participants to assess determinants of parental TM use for children during COVID-19 in Dire Dawa city, Eastern Ethiopia, 2023/24.

Variable	Category	Frequency	Percentage
**Information source about the TM**	Self	179	9.9
	Family, relative, friends, neighbors	703	38.9
	Health professionals	160	8.8
	Religious institutions	139	7.7
	Traditional healers	429	23.8
	Media	198	10.9
**Sources of TM used for their children**	Home	211	18
	Neighbors	375	32
	Traditional healers	584	50
**Is there anyone with TM skill in your home**	Yes	118	12.5
	No	823	87.5
**Have you had community based insurance coverage**	Yes	676	71.8
	No	265	28.2

### Need Factors

Among the major 601(53.1) purpose why parents seek TM for their children is to treat illness/symptoms. 611 (64.9%) of the parents perceives their children has poor health status prior to TM use; were half 468(49.7%) the children have 1–6 months duration of illness. However only half 464(49.3%) of the parents stated their children had improvement after TM use (Table 6).

**Table 6:** Need factor of the parents to assess determinants of parental TM use for children during COVID-19 in Dire Dawa city, Eastern Ethiopia, 2023/24.

Variable	Category	Frequency	Percentage
What was your purpose to use of TM for your child	1. To promote health	226	19.9
	2. To prevent illness	306	27
	3. To treat illness/symptom	601	53.1
How do you perceive your child’s heath prior to use TM	1. Poor	611	64.9
	2. Good	263	27.9
	3. Very good	67	7.1
Duration of illness	1. < 1month	74	7.9
	2. 1–6month	468	49.7
	3. ≥ 6mont	399	42.4
How would you state your child health condition after TM use	1. Improved	464	49.3
	2. Same	423	44.9
	3. Deteriorated	54	5.8

### Factors associated with parental TM use for children

After analyzing each independent variables of socio-demographic, need and enabling factors in bivariate analysis,variables that had a statistical significant association with parental TM use during Covid-19 at p-value less than 0.25 were entered into the final model for multivariate logistic regressions. Finally, variables with p-value less than 0.05 in the multivariate regression were declared as having statistically significant association with parental TM use during Covid-19.

On bivariate analysis factors such as relation of parent to child, educational status, marital status, religion, occupation, number of children in house hold, parental TM use for themselves, TM use for elder people, TM use for neonates & infants, easily accessible, safety and efficacy, cheap in price, information source of TM from family, relative, friends, neighbor as well as traditional healers, neighbors and home as source of TM and TM skill in the family were significantly associated with parental TM use for children during Covid-19.

After adjusting for potential confounder relation of parent to child, religion, parental TM use for themselves, easily accessible, safety and efficacy, TM skill in the family were found significantly associated with parental TM use for children during Covid-19 at p < 0.05).

Multivariate analysis indicated that family members who have sibling relationship to the children were 0.45 times (AOR: 0.45, 95% CI, 0.01–0.17) less likely to use TM for their children than mothers who are caregivers. Regarding parental religion children whose parents are Muslim religion follower were 0.11 (AOR: 0.11, 95% CI, 0.04–0.29) less likely to use TM for their children than orthodox religion followers.

Regarding parental TM use for themselves parents who had used TM for themselves had 6.55 (AOR: 6.55, 95% CI, 1.67–25.69) more likely to use TM for their children than those who had not used TM for themselves. Parents who have easily accessible to TM had 2.72 (AOR: 2.72, 95%CI, 1.16–6.40) more likely to use TM for their children than those who did not have. Also among another reason for preferring TM than conventional medicine caregivers who reported that TM has more safety and efficacy were 4.53 (AOR: 4.53, 95%CI, 2.3–13.61) more likely to use TM than their counterparties. Having TM skill in the household were 0.47 (AOR: 0.47, 95%CI, 0.19–0.84) less likely to use TM for their children than those without TM skill member in the house (Table 7).

**Table 7:** Bivariate and Multivariate Analysis to Identify Factors Associated with Parental TM use for Children during Covid-19 in Dire Dawa City, Eastern Ethiopia, 2023/24.

Variables	Ctategory		Parental TM use for Children Since COVID-19		COR(95%CI)	AOR(95%CI)	P value
			Yes	No			
Relation of parent to child	Mother		669(71.1%)	96(10.2%		1	
	Father		124(13.2)	9(0.9%)	1.97(.97-4.02)	3.16 (.77-13.03)	0.846
	Sibling		31(3.3%)	12(1.3%)	**.37(.18−.74)**	**.45(.01−.77)**	**0.004**
Educational status	Can’t read & write		66 (7%)	21 (2.2%)		1	
	Primary(1–8)		80(8.5%)	15(1.6%)	1.7(.81-3.55)	.91(.45-3.54)	.274
	Secondary(9–12)		416(44.2%)	57(6.1%)	2.32(1.32-4.08)	1.31(.34-3.6)	.862
	Higher		262(27.8%)	24(2.6%)	3.47(1.82-6.62)	.81(.21-2.02)	.663
Marital status	Single		32(3.4%)	16(1.7%)		1	
	Married		612(65%)	70(7.4%)	4.37(2.28-8.37)	19.71(2.71-43.1)	.178
	Divorced		97(10.3%)	15(1.6%)	3.22 (1.43-7.27)	.90(.11-7.05)	.652
	Widow		83(8.8%)	16(1.7%)	2.59(1.16-5.79)	.64(.20-2.03)	.173
Religion	Orthodox		248(30.4%)	38(4%)			
	Protestant		239(25.4%)	18(1.9%)	2.03(1.13-3.66)	1.63(.57-.4.63)	.954
	Muslim		337(35.8%)	61(6.5%)	**.85(.55-1.31)**	**.11(.04−.29)**	**.033**
Occupation	Government		427(45.5%)	54(%.7%)		**1**	
	Private		331(35.2%)	37(3.9%)	1.13(.72-1.76)	.88(44-1.78)	.404
	Daily Labor		41(4.4%)	14(1.5%)	.37(.19−.72)	.60(.21-1.72)	.765
	Others(specify)		25(2.7%)	12(1.3%)	.26(.12−.55)	.48(.15-1.56)	.248
Number of children	<3 year		508(54%)	93(9.9%)		**1**	
	3-5 year		316(36.1%)	24(2.6%)	2.41(1.50-3.86)	.82(.34-1.96)	.844
Have you used TM for your	Yes		707(75.1%)	109(11.6)	**2.26(1.07-4.75)**	**6.55(1.67-25.69)**	**0.013**
Self	No		117(12.4%)	8(.9%)		**1**	
TM use in the family members	Elderly people	Yes	388(41. %)	78(8.3)	2.24(1.49-3.38)	1(.50-2.02)	.203
		No	436(46.3%)	39(4.1)		1	
	Neonate& Infants	Yes	329(35%)	59(6.3%)	1.53(1.04-2.26)	.48(.52-2.04)	.086
		No	495(52.6%)	58(6.2%)		1	
Reason of preferring TM	Easily accessible	Yes	555(59%)	63(6.7%)	**.56(.38−.84)**	**2.72(1.16-6.40)**	**.025**
		No	269(28.6%)	54(5.7%)		**1**	
	Safety & Efficacy	Yes	162(17.2%)	53(5.6%)	**3.33(2.22-4.98)**	**4.53(2.3-13.61)**	**0.010**
		No	662(70.4%)	64(6.8%)		**1**	
	Cheap price	Yes	476(50.6%)	59(6.3%)	1.34(.91-1.98)	.62(.12−.56)	.219
		No	348(37%)	58(6.2%)		**1**	
Information source about the TM	Family, relative, friends, neighbor	Yes	643(68.3%)	55(5.8%)	.25(.17−.37)	.55(.02−.14)	0.062
		No	181(19.2%)	62(6.6%)		**1**	
	Traditional healers	Yes	398(42.3%)	64(6.8%)	1.29(.88-1.90)	1.23(.56-2.70)	0.962
		No	426(45.3%)	53(5.6%)		**1**	
Source of TM	Neighbour	Yes	540(57.4%)	49(5.2%)	.38(.26−.56)	.99(.48-2.06)	0.432
		No	284(30.2%)	68(7.2%)		**1**	
	Home	Yes	361(38.4%)	54(5.7%)	.09 (.04−.14)	1.16(.05-45)	0.733
		No	463(49.2%)	63(6.7%)		**1**	
TM skill in home	Yes		116(12.3)	91(9.7%)	**21.24(13.24-34.45)**	**.47(.19−.84)**	**0.015**
	No		708(75.2%)	26(2.8%)		1	

1= Reference, COR= Crude odds ratio, AOR= Adjusted odds ratio, 95% CI = 95% confidence interval

### Results for qualitative data

#### Socio-demographic background of study participants.

In this study, a qualitative part was conducted with an in-depth interview from a total of 16 participants. Among these eight were parents/care givers of the children, four herbalists, two religious/prayer, a bone settler and a tooth extractor were interviewed as key informants. Nine of the participants were females. The age of the participants ranges from 28–54years. Half, 8(50%) of the participants were orthodox religion followers (Table 8).

**Table 8:** Sociodemographic characteristic’s of study participants.

Code	Sex	Age	Address (Kebele)	Religion	Educational status	Occupation	TM Role
**P01**	M	42	Kezira	Muslim	Diploma	Teacher	Parent
**P02**	F	30	Gende Kore	Orthodox	Degree	Nurse	Parent
**P03**	F	44	Gende Gereda	Protestant	Degree	Private	Parent
**P04**	F	35	Wahil Kebele	Protestant	Degree	Private	Parent
**P05**	M	46	Gende Kore	Orthodox	Diploma	Private	Bone settler
**P06**	F	39	Wahil Kebele	Orthodox	Diploma	Farmer	Herbalist
**P07**	M	36	Kezira	Orthodox	Degree	Priest	Religious
**P08**	F	28	Wahil Kebele	Protestant	High school	Housewife	Herbalist
**P09**	M	50	Gende Gereda	Muslim	Diploma	Private	Herbalist
**P10**	F	38	Gende Gereda	Orthodox	Diploma	Daily Laborer	Parent
**P11**	M	47	Kezira	Muslim	Degree	Private	Herbalist
**P12**	F	54	Gende Kore	Orthodox	Illiterate	Housewife	Parent
**P13**	M	42	Gende kore	Protestant	Degree	Pastor	Religious
**P14**	M	51	Wahil Kebele	Orthodox	Diploma	Farmer	Tooth extractor
**P15**	F	40	Gende Gereda	Orthodox	Diploma	Private	Parents
**P16**	F	34	Kezira	Muslim	Degree	Teacher	Parent

Key: P- Participant F=Female M=male

### Emerged themes

Following repeated readings of the transcriptions and subsequent coding nine main themes were developed, with number of subcategories underneath each theme. The themes and subcategories are listed as follows (Table 9).

**Table 9:** Themes and subthemes that emerged after analyzing.

Themes	Subthemes
Commonly used TM Practices	#Herbal products #Religious Therapy #Bone settler #Massage
Ailments treated by TM	#Gastro intestinal #Mental and Behavioral problems #Respiratory # Musculoskeletal #Cardiovascular
Dosage and mode of TM application,	#Oral # Dermal #Topical
Preparation Preservation and Storage of Herbs	#Concotion #Crushing,
Parts of herbs used	#Leaves, #Stem and #roots
Indigenous TM knowledge acquisition & Transfer method	#Orally #By observation and Experience #Giftedness from God
Barriers for non-disclosure of TM use to HCP	#lack of awareness about potential side effect & interactions #Misperception that TM may be incompatible with conventional #drug which delay initiation of treatment #lack of trust in HCP and #Negative judgment from HCP
Reason for concomitant use	#Lack of improvements, #Coercion by others opinion, #Adverse reaction of TM, #worsening of the symptoms, #Ineffective conventional management, #Developing of complication and #Seeking conventional medicine as additional support.
Factors associated with parental TM use for children	#Socio demographic #Need factors #Enabling factors #Health care practice and experiences

### Common Type of TM Practices Used for Children and illness Treated by TM

This qualitative study finding revealed that participants commonly used to prefer TM practices for their children in almost for all types of illness such as respiratory, musculoskeletal, mental, behavioral, and spiritual. They use TM either for acute or chronic illness, minor to severe, curable or incurable, which they held belief in that it provides cure.

**A 34-year-old female; mother stated**

*“...Since immediately after the outbreak of COVID-19 pandemic was notified in our country, when almost all movements were restricted, my 4-year son got severe flu-like symptoms. I was highly suspicious that it may be symptoms of COVID-19. Previously I doubted the effectiveness of herbs and other TM approaches. I decided to consult both the herbalist and my close relative healthcare practitioner. Finally, I decided to give the herb. My son gets well after a couple of days. It is effective, and I was surprised”. (P16)*

**A 44-year-old mother reported;**

*“…Using TM becomes a habit of experience to use for our children for acute illness, which is not severe; it is usually a starter treatment option before seeking a health facility. If it is getting worse, it is time we seek a health facility. Almost all illness is managed easily with homemade remedies for older children, such as ginger, onion, lemon juice, and butter, and or a combination of these, as well as herbal products taken from herbalists*. *However, young infants and newborns usually taken to health facility as awareness from health care provider do not allow”*. (P03)

**A 42-year-old father stated**

“….*My kid gets twisting of his left leg while playing with his peers in the field. The injury seems minor and needs massage, not too severe to attend hospital. I talk to the bonesetter whom I know in person in our village. He checked the degree of injury, manipulated it manually, and told me to restrict his mobility. After two weeks he gets cured without deformity.”* (P01)

**A 40 year-old female parent said**

“… *As a family, we started attending available health facilities since she got ill 10 years back. The physician claimed it is a mental disorder and provided her with drugs. No definite cure was observed, and the neighbor’s told us to take her to the nearby local church for “tsebel”. Still there is recurrence but not a cure. We were following the regular praying programs in addition to tsebel. It is too difficult for the caregiver both mentally and economically, we sought all the available treatment option but still we are waiting for the will of God.”* (P15)

### Preparation, Preservation, and Storage of Herbs

Herbalist collects high-quality fresh herbal parts such as leaves, stems, and roots and inspects them visually for quality and efficacy. Parents themselves without any request for support usually make some of the herbs but herbalists prepare most of the herbs, and they give with detailed instructions for use. Decoction is the commonly used method to prepare the herbs. When prolonged duration of use needed, herbs are dried and ground thoroughly to powder form and mixed with water, oil, and organic honey during use.

Herbalists preserve and store the herbal products in the form of powder by keeping them in safe, dry, and airtight containers such as clay pots, bottles, and plastic containers. In some case, they hang it outdoors to preserve it. Herbs are prepared, preserved, and stored with a label according to their therapeutic purpose.

**A 47-year-old male herbalist stated**

*“….Herbal preparation in some cases is very simple, and we just instruct the parents or the customer to prepare by themselves at their own home. In fact, most of the remedies are popular and used by the majority of the community without consultation. However, in some cases we instruct parents to prepare on their own after they collect essential remedies either from their yard or from market. They should have to select fresh herbal parts, wash them for debris, and chop them into small pieces before mixing them with water. Then the mixture is boiled at lower boiling points (simmered) for half an hour to get the active ingredients. Then it will be strained and bottled if needed for a longer duration. I instruct them to drink until the symptoms subside. Here, cautiously, we instruct them to expose the ingredients for the proper duration of time to keep their efficacy”*. (P11)

**Also another 39 female herbalist said**

*“…..To have long-term use, I grind the herbs into a paste form that the patient can easily take by mouth. Plants with high efficacy were sorted and dried thoroughly before I ground them into a fine powder. Then the powder is mixed with a small amount of water, oil, or pure organic honey depending on the case of their illness and patient type. Then I cautiously measure the amount to be given to the children or the older user. Clear and detailed instruction is given to the user to have therapeutic efficacy”*. (P06)

**A 28 years old female Herbalist stated that**

*“….Herbs are prepared, preserved, and stored according to their therapeutic purpose, such as fever respiratory (common cold, asthma, and sinusitis), gastrointestinal (diarrhea, constipation, and intestinal parasite), antimalarial, skin, headache, and anti-pains. To prepare herbs for anti-pain, I usually mix different herbal products that have pain-relieving potency together. They are dried and stored safely. When the user comes with different headache types, I measure the mixture considering the age of the user and add hot water over 10 minutes to allow essential ingredients to be extracted. Then the patient is allowed to inhale the steam, which provides a cure for their illness.” (P08)*

*“….After herbal parts such as leaves, stem, and roots are collected and inspected visually for quality to ensure potency and efficacy, they will be dried and kept in dry containers such as clay pots, bottles, and plastic containers. Some of them may be grinded into powder forms to make them easy to use when the client comes. It has its own labelled containers for specific ailments that have similar backgrounds with plants names and their uses. In some cases I also hang the herbal product outdoors to dry. After that they are stored in air tight containers”*. (39 year old Female Herbalist, P06)

**A 50 year old Male Herbalist said**

*If a patient comes with a skin disorder, a topical herbal paste from “aloe vera” was applied by mashing the fresh leaves. To have a prolonged use from a dried extract, I add the herbs with clean oil to create the paste. Then the paste was applied directly over the affected area and covered with a clean cloth.* (P09)

### Dosage & Mode of application of Herbs

Herbal dosage for children is usually based on estimation and experience until symptoms are improved. There is no detailed consideration of weight or age. The duration is also determined by improvement of the symptoms.

**A 38-year-old mother said**

*“….The herbalist provided me with a dried part of leaves to be boiled in water and taken daily for a week: there is no detailed description for the duration of boiling. He provided me with an estimation to be given with half of a cup through the mouth. He only considered the physique and age*. *No other parameters are considered, like weight used in health facilities.”* (P10)

**A 50-Year-old Male Herbalist stated**

*“….I know the amount to be given for children from experience by considering the relative amount of adults. It actually depends upon the case, but there are no known complaints of adverse effects reported from the user. There is no precise dosage unit I use. However, in instances where there is no sign of improvement, I consider using either fresh form or a combination of available treatment modalities.”* (P06)

**A 54-year-old mother stated**

*“…The herbalist gave me a powder mixed with water to be given to my child with a cough by mouth until the symptoms subside for two weeks. My child’s condition is getting worse.He continued it for an additional week, but still no improvement. There was no dose adjustment, but he provided me a more mixed combination than the former herbal product. After three weeks of herbal therapy, I took my child to the hospital, and he treated with antibiotics for another 10 days. It was a time I noticed subjecting children to herbs is not a proper option*. (P12)

*“…Dosage of herbs was my big deal for a time. When I took my two-year-old child to the herbalist before two year, the amount of herb ordered is the same as that after 2 years for intestinal parasites and abdominal complaint. In addition, there is no detailed and clear-cut application order. They told you to mix with water to be given orally. In fact, it does not cured and I treated him at the health center. Thus, in children, preferring TM is questionable.”* (A 35-year-old Mother, P04)

### 4.11. Indigenous Knowledge Acquisition and Transfer Methods

The qualitative findings revealed that indigenous knowledge is acquired and transferred by experience and detailed observation, storytelling orally accompanied by personal dedication and commitment and giftedness from God.

**A 47 year old, male herbalist stated**

*“…I get the experience from my father. When I was child I was assisting him while he was collecting an herbal parts he uses for the treatment purpose in the nearby forest. When I was getting adult and his age is advancing, he told me to sustain his work and he started to explain specific herbs and their medical purpose, preparation, preservation and storage methods. Furthrer; more it needs deep-rooted expriences I was providing service while he was alive under his supervision. It is the source of income for him for decades. No one is frankly open to share his/her experience unless there is proximate kinship. However, in my view it is not simple task to transfer. It needs authentic dedication and scarification to serve and to become competent herbalist”*. (P11)

*“….In our village there are places where elder people gather and tell their indigenous knowledge and wisdom in the form of story. It is moment I was attracted and passionate with their healing capacity. The community provide them special credit for their action. I started following their footstep as an assistant during trip to forest. Prolonged exposure and the intuition to request for clarification with my good observational trend and practice as aid helped me to attain this skill after a long period. However; regarding TM knowledge acquisition and transfer there is no liner and formal way to do so. It is too broad and a difficult task without exaggeration”.* (50 year old herbalist; P09)

**Also a 51 year old male tooth extractor revealed that**

“…*There is no formal school to get such type of knowledge. It takes almost half of my age (30 years) to give the service independently in our village. Most of the neighbors get the service from our home. To the extent of my understanding there is no conventional treatment option in the past and even currently. In average we provide service to at least four children per day. It is tiresome know but previous I was assisting my dad Thus I get the experience from my dad 30 years ago. It doesn’t need special training but extensive years of observation and cautiously following the procedures.*” (P14)

A 42 year old pastor stated that *“…Nothing is from me I was created by the God almighty. I am a missionary of his will. I do nothing from myself. Everything is from divine power. The power to heal is the power of God. God is omnipotent. God can do all thing according to his will. To deliver from illness that declared has no cure is so simple for God. In the past, today and in the future God is all powerful. In spiritual realm to be enriched with spiritual giftedness you should be obedient to Gods will and living sin free life. I got the gift for free and giving it for free; it is Gods order. Nothing is from my side. Glory to almighty God. Many get relief from illness declared fatal. In some cases it requires earnestly fasting and prying”*. (P13)

### Reasons for Concomitant Use

Parents consider optional and or a combination of treatments for their children due to lack of improvements, coercion by others opinion, adverse reaction of TM, worsening of the symptoms, ineffective conventional management, developing of complication and seeking conventional medicine for additional support.

**A 30 year old mother stated**:

*“….Lack of improvement after repeated use of herbal products was one of the reason why I further sought heath care treatment. After I used traditional medicine for my child with cardiac case the symptom didn’t get improved over long period of time, the herbalist considered alternative change of the remedies but still no changes and symptoms worsen and my child becomes comatose. It a time I visited a nearby clinic in our village. The clinic owner provided me some sort of medication which alleviated the symptoms for time being but told me to have some further investigation to be done in Dire Dawa town. I did the investigation and they appointed me to have a follow up and he is now in good condition with the medication. I learned and changed my thinking that whatever the cost it charges on us visiting health care is premier option”*. (P02)

**A 34 year old mother also said that**

“…*When my 10 years on experienced asthma he don’t get relieve of it both traditionally and by conventional medicine. My neighbors and relatives are claiming the illness are originated from devil sprit and I also sought spiritual pray therapy. I worried a lot and decided to consider all the available option for cure. The doctor confirmed the diagnosis and told me all treatment outcomes. But the opinion of the relatives and urge to get cure makes me unstable. I tried all the three treatment options but know I already left the traditional one and using drugs provided by doctor with regular prayer*”.(P04)

**A 40 year-old female parent said**

*“…A year ago my 6 month old son developed difficulty of passing stool for two weeks. I talk to herbalist and he gave me some form of laxative herbs. After administering it my child developed persistent vomiting and abdominal colic and the symptoms become worsening. It was so annoying and I consulted nearby clinic were they treated my child accordingly”.* (P15)

**A 38 year old mother said**

*“…My son developed high grade fever. I try to apply cold compress and her grandmother told me to boil some sort of herbs and to be drink with milk. Simultaneously she applied butter and ash on his anterior head. Despite this supportive management the child condition was getting worse and I took him immediately to the hospital and they diagnosed and managed effectively”.* (P10)

**A 40 year-old female parent said**

*Two years ago my youngest daughter developed fever and uncontrolled shaking of body. I took her to near by the clinic but my neighbor told me to take to traditional healer. He applied fluid like oil with massage and she is getting good but I still took her to the hospital for further management. They treated with injectable drug and after a couple of days she become stable. (*P15)

**A 44 year old mother reported**

*“….When my Childs diabetes mellitus is uncontrolled and needs lifelong treatment. I try to rely on TM to cure. But still there is no definitive cure from both sides. Seeking both conventional and TM is for the reason to get additional support”. (*P03)

***A 38 year old mother said***

*“…After I visited hospital for my child with chronic arthritis the physiotherapist directed me to have massage by TM practioner. Combination of both approaches seems give best synergistic result but it remained only expectation”.(P10)*

**A 42 yeal old father stated**

*“….My 14 year’s old boy sustained dislocation of knee after motor vehicle accident. We took to bone settler he checked the site; aligned the bones and immobilized it. After the removal of the splint he developed contracture and mal-union which leads to admission to the hospital for further management”.* (P13)

### Barriers for Non Disclosure of TM use to Health Care Providers

Parents who used TM for their children don’t disclose their utilization to the HCP due to lack of awareness about potential side effect and interactions, misperception that TM may have incompatablity with conventional medicine which may delay initiation of treatment for my child with modern medicine, lack of trust in HCP and negative judgment from HCP.

*“…I had the experience of using the herbs for myself for a long time without any noticed adverse effects. So usually I apply same herbal products for my children with small dose. Also no TM practitioner told us it has adverse effects. So we assume it is side effect free and it doesn’t come to the topic of concern to deal with HCP”*. (A 44 year old mother, P03)

*“….In the past I used herbs for my child with diarrhea and it doesn’t get improved. Then I visited hospital after it was getting worse. The doctor asked the date of the onset and the duration of the illness. He noticed the lately seeking health facility. I told him everything honesty that we attended TM practitioner. The doctor discouraged me a lot and the situation was not good at that moment. It was dismissive incident after that I get afraid to disclose to them. Despite this there is also community level widely held perception that herbs are natural and free of any side effects”.* (A 38 year old mother, P10)

*“….I gave herbs to my child but there is no improvement then I took her to the HCP.I have a fear that if I told the doctor they will delay initiation of modern medicine which worsen and further threaten my child’s life. So I preferred not to disclose it. However; I assume it is wrong approach. I have the opportunity to have free dialogue with them which may not jeopardize my Childs health status. Thus fear of negative judgment from HCP hindered me not have honest conversation”. (*A 30 year old mother *P02)*

*“….I encountered situation where I was not get enough information from the HCP. No improvement was obtained and I used alternative. Their approach is of judgmental type and they do not pay value to your concern. Sometimes they do not include you in the decision making process. They made decision by their own self with their preoccupied intuition”. (*A 42 year old father, P01)

**Factors Associated with parental TM use for Children**

### Socio-demographic Factors

*“I use the herbs because they are easily available with in our rural areas. No more time and cost is needed to get the herbal products. The TM practitioners are with in our community and in some cases they are available at home level*.” (A 42 year old father, P01)

“*I consider to use TM as we have financial constraints, conventional drugs are expensive in terms of cost and time to get them. So as option we have to consider to use TM. In fact it is more common among those who are less educated but those who live in urban and have good income don’t mostly relay on TM*”. (A 28 years old female Herbalist, P08)

“*Healing power is among the spiritual giftedness. It is a part of our religious belief and free of cost. Different parents usually relay on prayer after they medically claimed no cure. The Divine intervention is required”.* (A 36 year old priest, P07)

### Enabling factors

*“We can’t affords the high cost of modern drugs. We sometimes get TM service for free or even when you pay it is very minimal. It is very cheap”*. *(A 36 year old priest, P07)*

*I can easily afford and access when they are needed. Almost they are free of the cost. It cost us the travel cost to the health facility*. (A 42 year old father, P01)

### Need Factors

*“…As a parent we consider the severity of the case if mild and resembles resolve with home remedies we may not seek conventional therapy*.” (A 44 year old mother; P03)

*“…Using TM become a habit of experience to use to our children for acute illness which is not severe. It is usually a starter treatment option before seeking health facility. If it is getting worse it is a time we seek health facility.”* (A 44 year old mother, P03)

*“….When my Childs diabetes mellitus is uncontrolled and needs lifelong treatment. I try to rely on TM to cure. ”.* (A 44 year old mother, P03)

### Health care practice and experience

*“…I had the experience of using the herbs for myself for a long time without any noticed adverse effects. So usually I apply same herbal products for my children with small dose”.* (A 44 year old mother, P03)

*“…After I visited hospital for my child with chronic arthritis the physiotherapist directed me to have massage by TM practitioner. The TM Practitioners can provide service to illness considered ineffective by modern medicine”* (A 38 year old mother, P10)

*“….I encountered situation where I was not get enough information from the HCP. No improvement was obtained and I used alternative. (*A 42 year old father, P01)

### Triangulation of quantitative and qualitative

## Discussion

Traditional medicine continues to serve as the primary treatment modality for children, and its popularity among parents is on the rise. In this study, the prevalence of parental traditional medicine use for children was 87.6%. This study is in line with a population-based survey conducted in Motta town, where TM utilization for children was found to be 88.2% [6]. Similarly, this study was in line with the previous studies conducted in Tole district (85.9%) [37]. However, it is lower than the study finding in the North, Mecha district, 90.3% [5]. This variation may be attributed to study setting differences, where some parts of the country use more TM than other parts.

In this study, the most commonly used TM therapies were herbal medicine (67.1%) and religious therapy (27.7%). This study is in line with research done in Motta town and Tole district [5,6]. However, the findings from a study done in Fagita Lekoma Woreda, Northwest Ethiopia, found that herbal medicine and bone setters are the common TM parents used for their children [35]. This is almost similar with other findings but variation in bone settler may be attributed to the fact that bone settler is used for broad scope of both medical and surgical musculoskeletal management such as sprain, strain, fracture and dislocation as well as joint pain and arthritis. The qualitative finding of this study also revealed that parents use herbal products for almost all types of illnesses, such as respiratory, musculoskeletal, mental, behavioral, and spiritual.

Regarding the household use of TM, it is used for almost all family members, where children and adults share the majority, 857 (44.9%) of the TM use. The majority, 708 (75.2%), of the TM was taken by oral route. The quantitative finding of this study was in line with the study conducted in the Gera district [41] and Gulele in Addis Ababa, Ethiopia, where the oral route was found to be the major route of administration, followed by dermal [42].

Respiratory illness (27.6%), followed by musculoskeletal, was the major reason for parental TM use for children. However, the study in Motta town found that headache and gastrointestinal tract (GIT) were the top reasons why parents used TM for their children. Also, a study conducted in Lagos, Nigeria, revealed that herbal products are used for the treatment of common ailments such as diarrhea, skin disorders, abdominal cramps, jaundice, fever, malaria, insomnia, convulsions, and weight loss in neonates and infants less than 6 months [43]. This variation from study to study may be attributed to the different backgrounds of study participants in different settings where the use of and the scope of TM practice is broadly used for preventive, curative, and therapeutic purposes.

The qualitative findings of this study revealed that decoction is the commonly used method to prepare the herbs. When prolonged duration of use is needed, herbs are dried and ground thoroughly to powder form and mixed with water, oil, and organic honey during use. This finding is in line with a study done in Haramaya, Eastern Ethiopia, where water is the most preferred solvent added during the preparation of medicinal plants [44]. Similarly, the study in Harla and Dengego in Eastern Ethiopia found that concoction is the most common method of herbal medicine preparation [45].

Regarding herbal preservation and storage the qualitative finding of this study indicated that the herbalist preserves and stores herbal products in the form of powder by keeping them in safe, dry, and airtight containers such as clay pots, bottles, and plastic containers. In some cases they hang it outdoors to preserve it. Herbs are prepared, preserved, and stored with a label according to their therapeutic purpose. These findings are in line with the study done on the demand for fresh herbal products, which are difficult, and they preferred preserved, properly, and stored herbs [46]. However, the finding of this study contrasts with the study done in Boricha district, where fresh forms of herbs are used to treat different ailments than the preserved and stored herbs. This difference might be due to the study place, where those who live in rural areas have easier access to herbal products than urban residents might.

The qualitative findings of this study indicated that herbal dosage for children is usually based on estimation and experience until the symptoms get improved. There is no detailed consideration of weight or age. Duration is also determined by improvement of the symptoms. This is similar to findings where usage depends on ontological knowledge, and the dosage of TM often lacks pragmatic consideration of the user’s personal health profile (24).

The qualitative findings revealed that indigenous knowledge is acquired and transferred by experience and detailed observation, storytelling orally accompanied by personal dedication, commitment, and giftedness from God. This is similar to the finding in Gamo people (58).

The quantitative study findings indicated 71.9% of the parents who used TM also visited a health facility for the same illness after using TM. 90.6% of the parents who visited the health facility have concomitant use. No improvement by TM, and the healthcare provider did not request pre-hospital TM usage, was the main reason for concomitant use. Similarly, the qualitative study indicated that lack of improvements, coercion by others opinions, adverse reaction of TM, worsening of the symptoms, ineffective conventional management, developing complications, and seeking conventional medicine for additional support were among the reasons for concomitant use. There is no study done on factors for concomitant use to compare the study findings.

The quantitative findings revealed that 98.1% of those parents who visited the health facility did not disclose their TM use for their children to the HCP irrespective of the request by the healthcare provider. No request about TM use by HCP, fear of negative attitude, and TM practitioners not allowing are among the factors why parents don’t disclose the pre-hospital TM usage. The qualitative finding of this study also reported that lack of awareness about potential side effects and interactions, misperception that TM may have incompatibility with conventional medicine, which may delay initiation of treatment for my child with modern medicine, lack of trust in HCP, and negative judgement from HCP were among the barriers that hindered parents from using TM with HCP.

After adjusting for the confounding factor, multivariate analysis indicated that family members who have sibling relationships with the children were 0.45 times (AOR: 0.45, 95% CI, 0.01–0.77) less likely to use TM for their sibling than mothers who are cares. This may be attributed to the fact that the majority (81.3%) of the care providers in this study were mothers who experienced TM use for themselves (86.7%), which pushed them to use TM for their children. The qualitative part of this study also revealed that participants had experience using TM for their children from their own experience.

Among other sociodemographic factors that were statistically associated with parental TM use for children was parental religion. Children whose parents are followers of the Muslim religion were 0.11 (AOR: 0.11, 95% CI, 0.04–0.29) less likely to use TM for their children than orthodox religion followers. This may be due to the reason that TM is part of religion used for different acute and chronic illnesses in Orthodox and Protestant religions. The study is in line with the qualitative finding of this study, where healing power from religion is considered as giftedness.

Regarding parental TM use for themselves, parents who had used TM for themselves were 6.55 (AOR: 6.55, 95% CI, 1.67–25.69) times more likely to use TM for their children than those who had not used TM for themselves. This finding is consistent with the study done in southern Arizona, where parents who had used TM for themselves had a high probability of using TM for their children [47](64). Also, a study in Canada revealed that parents who used CAM for themselves had 9.4 more odds to use CAM for their children [48]. Similarly, findings from the USA revealed that parental CAM use is a significant factor in the use of CAM for children [49].

Parents who had easy access to TM were 2.72 (AOR: 2.72, 95% CI, 1.16–6.40) times more likely to use TM for their children than those who didn’t have it. This finding is congruent with the study done in Motta town, northern Ethiopia, where those parents who perceived TM as easily accessible were 2.97 times more likely to use TM [AOR = 2.97(P.0.004, 95% C.I.1.42–6.18)] for their children when compared to those who perceived TM as not accessible. Similarly, the study in Fagita Lekoma Woreda also indicated that parents who had access to TM were 2.21 times more likely to use TM for their children than those who had no access (AOR = 2.21, 95% CI = 1.23–3.98). This may be due to the socioeconomic status of the parents, where the majority of participants can utilize products with low cost within their setups.

Also among the other reasons for preferring TM over conventional medicine, caregivers who reported that TM has more safety and efficacy were 4.53 (AOR: 4.53, 95% CI, 2.3–13.61) more likely to use TM than their counterparts. This is in line with a study done in Motta town, where the perceived effectiveness of TM was another factor significantly related to parental traditional medicine utilization. Parents who perceived TM as effective were 2.48 times more likely to use it for their children than those who perceived it as not accessible [6]. Similarly, a study in Addis Ababa revealed that efficacy was one of the benchmark criteria to choose either TM or a conventional drug [34]. This finding is also supported by the qualitative finding of this study.

Having TM skill in the household was 0.47 (AOR: 0.47, 95% CI, 0.19–0.84) less likely to use TM for their children than those without TM skill members in the house. The possible explanation for this might be due to the fact that there are few TM practitioners within the community where TM users seek service outside of their home within their community.

## Conclusion and recommendation

### Conclusion

The prevalence of parental TM practice during Covid-19 for children was high (87.6%) indicating that TM is significantly contributing to the public health for promotion, prevention and treatment of different ailments. Herbal medicine was the most commonly used TM. In this study relation of parent to child, religion, parental TM use for themselves, easily accessible, safety and efficacy, TM skill in the family were found significantly associated with parental TM use for children during Covid-19.This study revealed high concomitant utilization (90.6%) and high non-disclosure rate (98.1%) of TM use to HCP. Non-disclosure were due to lack of awareness about potential side effects and interactions, misperception that TM may have incompatibility with conventional medicine, lack of trust in HCP, and negative judgement from HCP were among the barriers that hindered parents from using TM with HCP. Additionally, dosage of TM usually lacks pragmatic consideration of children’s characteristics and usually based on ontological knowledge until the symptoms get relieved.

### Recommendation

Based on this study we would like to recommend the Ethiopian minister of health and other concerned bodies to strengthen implementation of TM integration policy with modern health care system and utilize various strategies in controlling illegal TM trader. The health workers also need to provide health information for parents or guardians regarding the TM and incorporates TM intake history before diagnosis. Further research using clinical based experimental design with representative sample focusing specific herbal therapeutic use. Parents (caregivers) need to be aware of the effects of TM and refrains from using TM from illegal practitioners.

### Study strengths and limitations

The study used mixed method approach which explore special consideration during provision of service for special children (dosage, safety and efficacy), reason for concomitant use, indigenous TM knowledge acquisition and transfer methods, preparation, preservation and storage of herbal products which was not addressed by the quantitative part of the study. However, being reliance on caregiver report introduce recall bias and social desirability biases.

## Supporting information

S1 File Quan_questionnaire. (DOCX)

## Abbreviations

AOR: Adjusted Odds Ratio
CBHI: Community Based Health Insurance
CI: Confidence Interval
DDU: Dire Dawa University
ICU: Intensive Care Unit
FGD: Focused Group Discussion
HCP: Health Care Provider
GIT: Gastro Intestinal Tract
NICU: Neonatal Intensive Care Unit
TCAM: Traditional Complementary and Alternative Medicine
TM: Traditional Medicine
SSA: Sub-Saharan Africa
SPSS: Statistical Package for Social Science
