# Supplementary material for: Determinants of parental traditional medicine use for children during COVID-19 in Dire Dawa city administration, Eastern Ethiopia, 2023/24: Mixed community based cross-sectional study design
Source: PLoS One. 2026 Jul 30;21(7):e0354889. doi: 10.1371/journal.pone.0354889 (PMC13422885; doi:10.1371/journal.pone.0354889)
Supplement: S1 File — (DOCX) [file pone.0354889.s001.docx]

## Annex II: Data Collection Tool for Quantitative study

This tool was prepared for the collection of socio-demographic, health care practice & experience, enabling and need factor related information that are important for the assessment of determinants of parental TM use for children during covid-19 in Dire Dawa city, Eastern Ethiopia, 2023/24.This information will be collected by interviewing parents of children by trained data collectors.

Date:____________ Code:____________ Cluster:__________ Kebele___________

| **Part I: Socio-demographic Factors** | | |
| --- | --- | --- |
| No | Question | Response |
| 101 | Age of the child(days/month/years) | ________________ |
| 102 | Age of the parent | _______years |
| 103 | Relation of the parent to the child | 1. Mothers  2. Father  3. Sibling  4. Other(specify)__________________ |
| 104 | Marital status | 1. Single  2. Married  3. Divorced  4. Widow |
| 105 | Religion | 1. Orthodox  2. Protestant  3. Muslim  4. Others(Specify)________________ |
| 106 | Educational status | 1.Can’t read and write  2.Primary(1-8)  3. Secondary(9-12)  4.Higher |
| 107 | Occupation | 1.Government Employee  2.Private Employee  3.Daily Labor  4.Others(specify)_________________ |
| 108 | Residency | 1.Rural  2. Urban |
| 109 | Monthly income | ____________ETB |
| 110 | Number of children below 18 years | 1.>3  2. 3-5  3. >5 |
| **Part II: Health care Practice & Experience** | | |
| 201 | Have you ever used TM for yourself | 1. Yes 2. No |
| 202 | For whom TM is used in your family(Select all that apply) | 1. Elderly people 2. Children and adults 3. Neonates and Infants 4. Pregnant 5. Other(specify)_____________ |
| 203 | What was your reasons for preferring TM than modern medicine | 1. Easily accessible and affordable 2. Safety and efficacy 3. Cheap in price 4. Having low income 5. Dissatisfaction with modern medicine 6. Side effects fear of modern medicine 7. Difficulty in accessing health care facilities   Other (Specify)________________ |
| 204 | Have you ever used TM since Covid-19 outbreak for your child | 1. Yes 2. No……( Q218) |
| 205 | If your response is yes to question 203  when was the last time you applied TM for your child | 1. Last week -month  2. Month- Last six month  3. 6-12months  4. A 1-2 years  5. Before two years |
| 206 | If your response was yes to 203 what type of TM you had used for your child | 1. Religious/prayer therapy 2. Herbal Products 3. Bone settlers 4. Massage 5. Tooth extractor 6. Trained traditional birth attendant 7. Any others (specify)_______________ |
| 207 | Which route was used to administer TM | 1. Oral 2. Topical 3. Nasal 4. Rectal 5. Others(Specify)________________ |
| 208 | For what type of illness you preferred to use TM for your child | 1. Covid-19 2. Fever 3. Headache 4. Psychological 5. Musculoskeletal 6. Cardiac 7. GIT 8. Respiratory 9. Other(Specify)______________ |
| 210 | Have you visited health facility for same illness after using TM | 1. Yes 2. No ( Q214) |
| 211 | For what reason | 1. No improvement 2. Develop side effect 3. Other(specify) |
| 212 | If your response is yes to question 209, does the health care provider requested you about pre-hospital TM use | 1. Yes 2. No |
| 213 | If you visited health facility and not requested about pre-hospital TM use by HCP, have you disclosed the use irrespective of the request? | 1. Yes 2. No |
| 214 | Have you used both conventional & TM drug for your child at same time | 1. Yes 2. No |
| 215 | Have you ever told about side effect of TM you used for your child by the provider of TM | 1. Yes 2. No |
| 216 | How do you rate your level of satisfaction of TM use to your child | 1. Completely dissatisfied 2. Somewhat dissatisfied 3. Neither satisfied nor dissatisfied 4. Somewhat satisfied 5. Completely satisfied |
| 217 | How do you state your level of efficacy of TM used | 1. Excellent 2. Good 3. Poor |
| 218 | Would you believe TM is side effect free? | 1. Yes 2. No 3. Assumed to be side effect free |
| 219 | Would you thing it is ethical and legal to discuss TM use with your health care provider? | 1. Yes  2. No |
| 220 | Would you recommend TM to be used by others? | 1. Yes 2. No |
| 221 | What would you think to be barrier to not disclose your TM usage status to your HCP | ___________________________ |
| **Part III: Enabling Factors** | | |
| 301 | What are your sources of information about the TM | 1. Self 2. Family, relative, friends, neighbors 3. Health professionals 4. Religious institutions 5. Traditional healers 6. Media 7. Any other Specify_________________ |
| 302 | Sources of TM | 1. Home 2. Neighbors 3. Traditional healers 4. Other(specify)__________________ |
| 303 | Is there anyone with TM skill in your home | 1. Yes 2. No |
| 304 | Have you had community based insurance coverage | 1. Yes  2. No |
| **Part IV: Need Factors (**for those who used TM during Covid-19**)** | | |
| 401 | What was your purpose to use TM for your child | 1. To promote health  2. To prevent illness  3. To treat illness/symptom  4. Others?(specify)_______________ |
| 402 | How do you perceive your child’s heath prior to use TM | 1. Poor  2. Good  3. Very good |
| 403 | Duration of illness | 1. < 1month  2. 1-6month  3. ≥6mont |
| 404 | How would you state your child health condition after TM use | 1. Improved  2. Same  3. Deteriorated |

## ANNEX V: Data Collection Tool for Qualitative data

**In-depth interview questions for parents of children who used TM during COVID-19**

1. Would you tell me about TM Use in your community (kebele)

(Probe; to their own personal lived experience)

- Use for themselves, to whom TM use was allowed, for what purpose,
- Types of TM used and illness, last time used, route of administration, dosage, source of TM & information,
- Visiting health facility for treatment, reason, concomitant use,
- Disclosure of TM, request of TM use by health care provider, Disclosure without request by health care provider, barriers and facilitators of disclosure
- Safety parameters (Would you belief TM is side effect free? Told about side effect of TM you used by provider, how you check expiration date, storage place and condition
- How do you state your level of satisfaction and health status of your child after TM? Do you recommend TM to be used by others? why

1. **Questions for Key informants** (TM healers/Practitioners) Such as religious/prayer therapy, herbalist, bone settlers, tooth extractor, trained traditional birth attendant
2. Would you tell us your lived experience of serving community with your special skill and knowledge**? Probe**

- **Y**ears of experience, special consideration during provision of service for special population like infants, children, pregnant and elderly people (focus on children dosage, side effect and its management, way of detecting side effect)
- **For Herbalist** How you prepare, store, and preserve herbal products (Also observe environment, storage equipment, and date of production) and consideration of customer profile age, illness status,
- How you acquired your skill? to who and how you would like transfer the skill to serve the community)
- Any recognition, promotion, support and training from concerned body
- Opportunities and challenges while serving the community
- Would you thing integrating traditional medication with conventional medicine will enhance your service? (Probe **licensure)**
